# Social Determinants of Health Impact Cardiovascular Outcomes in Hospitalized Older Adults

**DOI:** 10.1093/geroni/igaf122.3654

**Published:** 2025-12-31

**Authors:** Vania Remenik-Zarauz, Ruth Pius, Malay Das, Andrea Reyes-Vega

**Affiliations:** Lincoln Medical and Mental Health Center, Bronx, New York, United States; Lincoln Medical and Mental Health Center, Bronx, New York, United States; Lincoln Medical and Mental Health Center, Bronx, New York, United States; University of Louisville, University of Louisville, Kentucky, United States

## Abstract

Social determinants of health (SDoH) have been reported to exert a significant impact on cardiovascular risk. However, there is limited research on the role of SDoH in cardiovascular outcomes in hospitalized older adults. This study aims to examine the impact of socioeconomic status, housing and healthcare access as SDoH on in-hospital outcomes among older adults with acute decompensated heart failure (ADHF), using the National Inpatient Sample (NIS) database from 2016 to 2020. We analyzed 9.2 million ADHF patients stratifying them according to age 65-79 (53%) and ≥80 (47%). The outcomes studied were death, cardiac arrest and cardiogenic shock. A multivariable logistic regression model was used (p < 0.05 was considered statistically significant). Within the 65-79 subgroup, all examined SDoH were statistically significant risk factors for cardiogenic shock (p = < 0.001) and death (lack of health insurance and homelessness p = < 0.001, poor income p = 0.008). Interestingly, homelessness was the only SDoH significantly associated with cardiac arrest in this age group (p = < 0.001). Within the ≥80 subgroup, lack of health insurance was the only SDoH associated with death (p = < 0.001), while cardiogenic shock and cardiac arrest were not significantly associated with any SDoH. These findings reinforce the role of social disparities in adverse outcomes among older adults, highlighting age-related vulnerabilities and the need for interventions addressing both socioeconomic and pathophysiologic mechanisms of disease.

